# The Application of Angulated Screw-Channels in Metal-Free, Implant-Supported Restorations: A Retrospective Survival Analysis

**DOI:** 10.3390/ma14227006

**Published:** 2021-11-19

**Authors:** Edoardo Rella, Paolo De Angelis, Giovanni Damis, Antonio D’Addona, Paolo Francesco Manicone

**Affiliations:** Department of Head and Neck and Sensory Organs, Division of Oral Surgery and Implantology, Fondazione Policlinico Universitario A. Gemelli IRCCS—Università Cattolica del Sacro Cuore, 00168 Rome, Italy; dr.paolodeangelis@gmail.com (P.D.A.); giovannidamis@gmail.com (G.D.); antonio.daddona@unicatt.it (A.D.); paolofrancesco.manicone@unicatt.it (P.F.M.)

**Keywords:** implantology, screw-retained, zirconia

## Abstract

Angulated screw channels (ASC) allow the clinician to reposition the access hole of screw-retained restorations, improving the design of the rehabilitation and the esthetic outcome. Few clinical studies are available on the efficacy of these restorations, especially at longer follow-ups and with a large number of subjects. The objective of this study was therefore to retrospectively evaluate patients rehabilitated with screw-retained restorations using ASC. The time of delivery and their adherence to the maintenance program was obtained, as well as the characteristics of the restoration and of the patient’s occlusion; a Kaplan–Meier survival curve was then built to investigate the success rate of these restorations and the effects of several variables were evaluated with a Cox model. A total of 105 subjects and 162 implants were enrolled in this study; after 42 months a success rate (92%) similar to what is reported for conventional screw-retained restorations was encountered. Monolithic zirconia restorations (*n* = 52) had a higher success rate (95%) when compared to partially veneered restorations (*n* = 53), which suffered a higher number of complications (90%). The other variables had no statistically significant effect. Implant supported prostheses adopting ASC provide a favorable outcome both in the posterior and anterior regions and can therefore be adopted to treat cases where the implant angulation is unfavorable for a conventional screw-retained prosthesis.

## 1. Introduction

Implant supported restorations have long been the standard of care for treating partial edentulism [1,2] given their major advantages over other rehabilitative options and are now well documented in the literature [3]. According to recent systematic reviews, implant-borne reconstructions exhibit excellent survival rates, similar to tooth-supported restorations, reaching 95% at 5 years follow-up [4]. The clinical success of restorations does not only amount to the survival rates, but also to the frequency of both mechanical and biological complications that can occur during the life of the restoration. One of the main leading causes of failure for implants is still peri-implantitis, and choosing the correct design of a restoration is paramount to achieve a stable result, as even the emergence profile can influence the development of peri-implant diseases [5,6].

One of the major debates around implant-supported restorations, in an effort to improve the success of those restorations, is related to the fixation method between the restoration and the implant fixture; when a restoration is planned the clinician can choose between several retention systems with the two major options being cement-retained restorations and screw-retained restorations [7]. Cement-retained restorations were initially adopted for single unit restorations, where the restoration itself was cemented on pre-fabricated components [8]. They most closely resemble tooth-supported restorations, both in clinical and technical procedures; therefore, they might be easier to fabricate and manipulate. They have the advantage of being slightly more aesthetically pleasant, as no access hole is required, and have a higher chance of providing a true passive fit, as cement can compensate for some of the discrepancies that inevitably originate from several clinical and laboratory steps. Nonetheless, the removal of these restorations is difficult and may even be impossible without destruction of the restoration. The major flaw of cement-retained restoration is the risk of leaving traces of cement in the peri-implant sulcus; given the low resistance offered by peri-implant tissues during cementation, the cement can flow well below the abutment, even reaching the implant fixture. This creates a rough and porous surface where plaque can easily be deposited and has been proven to be a leading cause of peri-implantitis in cement-retained restorations [9,10,11], and the many methods that have been proposed to reduce the probability of residual cement do not have adequate efficacy.

On the contrary, screw-retained restorations, initially only adopted for full-arch restorations [12], can be much more easily retrieved if a complication develops [13,14]. They are also quite unlikely to be subject to biological complications, provided that a good fit is obtained. They also have their shortcomings: the position and angulation of the implant fixture is much more critical with this type of restoration as the screw access hole should be positioned on the palatal or occlusal surface, which can be achieved only if the implant position was adequately planned. Therefore, conventional screw-retained restorations are usually contraindicated when the access hole would be positioned on the vestibular surface of the restoration, which would produce a poor esthetical outcome. Additionally, they might present a higher risk of mechanical complications as the thickness of the restorative material around the access hole can be quite irregular. Some authors have reported on the possibility of technical complications such as chipping of the veneering ceramic and loosening of retaining screws [15].

With the introduction of Angulated Screw Channels (ASC), the access hole can be relocated to the lingual face of an anterior tooth or to the occlusal surface of a posterior tooth, also controlling the thickness of the material [16]. This concept alters the angulation of the screw channel within the restoration, while maintaining the abutment and the fixture alignment. Several restorative systems currently apply this concept, which can correct and accommodate a divergence up to 30 degrees from the implant axis [17].

ASCs can be applied to Porcelain Fused to Metal and, thanks to novel computer-aided design and computer-aided manufacturing (CAD-CAM) systems, to Zirconia based restorations, both monolithic (completely made in zirconia, and then superficially colored [18]) and veneered (where a cutback is added in the design phase to provide space for subsequent veneering with ceramic [19]) [20].

Few clinical reports have reported on the efficacy of ASC in single crowns (SC) and Fixed Partial Dentures (FPD), but these preliminary results show that these restorations, even if at a 1 year follow-up [21,22], can provide a satisfactory result. Some in vitro studies have reported that as the channel is not perpendicular to the implant, which may receive off-axis loading, the rate of mechanical complications could possibly be higher than conventional, screw-retained, FPD. Additionally, as the force that generates the torque in the fixating screw is not perpendicular to the screw itself, but is positioned at an angle, theoretically a lower percentage of that force is transferred, resulting in a lower preload [23,24,25].

The objective of this article is therefore to analyze the success rate of screw-retained, zirconia-based restorations with a survival analysis, and to underline if a different design, either monolithic or partially veneered, can influence the outcome of the restoration. The null hypothesis was that monolithic restoration and partially veneered restorations had the same success rate.

## 2. Materials and Methods

This study is a retrospective, longitudinal clinical review of cases treated in dental private practices by three practitioners experienced in the field of implant prosthetics rehabilitations.

A comprehensive database was required, which was obtained from the personal records of these patients; in order to be included in the present study patients had to be 18 or older at the time of implant positioning, to exclude cases where a premature rehabilitation might have jeopardized the result, and had received a full-ceramic screw-retained restoration with the application of ASC.

Patients with a history of intravenous or oral bisphosphonates, uncontrolled diabetes, or affected by uncontrolled periodontitis were excluded from the study. All patients had been treated with the following protocol and American Dental Association (ADA) guidelines were followed during all clinical steps.

Before the surgical phase, all patients were submitted to a preliminary preparatory phase including mechanical debridement with ultrasonic and, if needed, manual instruments so that no pocket with a periodontal probing depth ≥ 5 mm was present.

Implant fixtures (Straumann^®^, Biomet 3I^®^, Palm Beach Garden, FL, USA) were placed under local anesthesia, after elevating a full thickness flap, following the manufacturer’s guidelines. Preoperative prosthetic planning was performed for all cases.

Four months after implant positioning, a conventional impression using a custom tray and a polyether (Impregum Penta Soft Quick Step MB, 3M ESPE, St. Paul, MN, USA) impression material with the open-tray technique, or a digital impression with a digital scanner (Trios 3, 3SHAPE, Copenhagen, Denmark) was taken. In the first case, an impression of the antagonist arch was taken with an impression material (Position Penta, 3M ESPE, St. Paul, MN, USA) and the bite was registered using hard wax. (Moiko Beauty Pink Wax, Integra Miltex, York, PA, USA). The impressions were poured in stone and the models were scanned (Sinergia Scan, Nobil Metal, Villafranca D’Asti, Italy).

**Figure 1:**
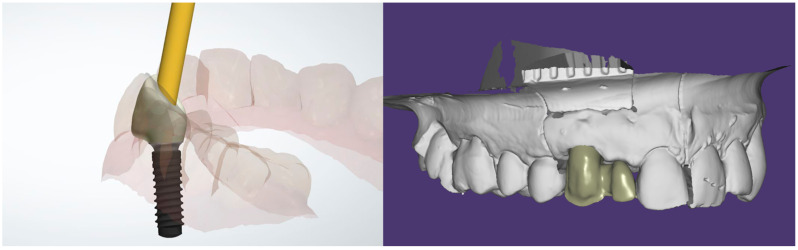
The digital project of a monolithic zirconia crown and a zirconia framework later veneered (0.8 mm cutback).

**Figure 2:**
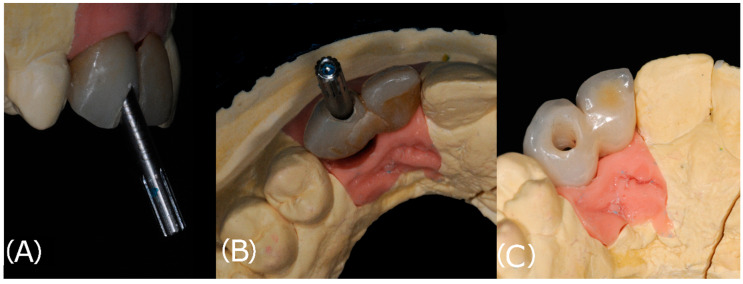
(**A**): The inclination of the implant fixture determined an unfavorable position of the access hole in the provisional restoration. (**B**): The access hole as seen from the occlusal surface. (**C**): Thanks to the application of ASCs in the definitive restoration, the access hole was moved to the palatal surface.

The restorations were either designed as Monolithic Zirconia Restorations (MZR) or as Partially Veneered Restorations (PVR), in which case a vestibular cutback was included in the design. (Figure 1) Following the CAD-CAM processes, where the screw channels were designed as angulated(>15 degrees), the restorations were finished either by adding a vestibular veneer (PVR) or by a surface characterization (MZR). Surface characterization was done following the indications provided by the manufacturer, applying the necessary coloring solutions with a brush with delicate movement from the occlusal to the cervical zone (Figure 2).

High-translucency (HT) zirconia was utilized, in the form of inCoris TZI (Dentsply Sirona, Charlotte, NC, USA) and Biodynamic Multilayer 1200/600 Mpa Progressive (Biodynamic, Lesignano de Bagni, Italy). The former is an HT zirconia with flexural strength > 900 MPa, while the latter is a more innovative material that presents higher flexural strength in the cervical region (1200 MPa), where more mechanical strength is needed, and lower flexural strength (600 MPa) in the incisal region, where more translucency is preferred.

**Figure 3:**
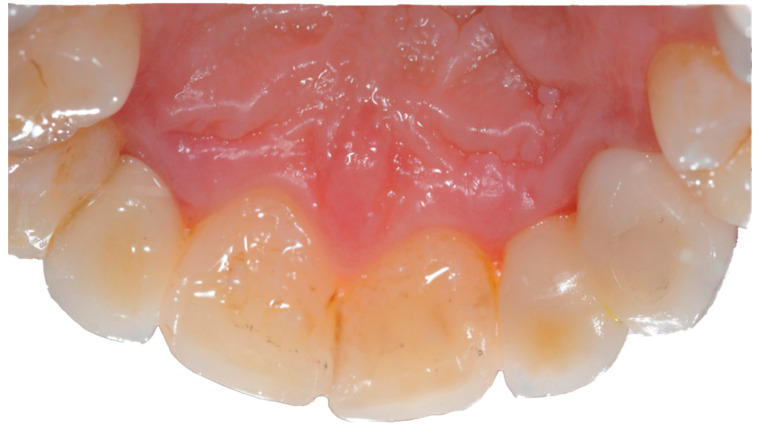
After screwing the restoration (tooth number 1.2 and 1.3) to the implant fixture the access hole, moved to the palatal surface, was sealed.

The FPD were then bonded to the abutments (Link-In System, New Anchorvis, Calderara di Reno, Italy) and delivered to the clinician. The restorations were then tried, and after adjusting the bite, were tightened to the implants (25 N/cm). The clinician then sealed the access hole with polytetrafluoroethylene tape and composite resin (Figure 3). An intra-oral radiograph was taken to assess the seating of the FPD.

The date of delivery of the restoration, the design (either monolithic or partially veneered) and type of restoration (either an SC or an FPD), the condition of the opposing tooth, the position of the restoration, and the anagraphic characteristics of the patient (sex and age) were recorded and inserted in an electronic spreadsheet.

Patients were then inserted in a maintenance protocol and were therefore recalled every 4–6 months according to their risk status. At each follow-up appointment the condition of the restoration was assessed, and any complication was recorded.

The study was conducted in accordance with the Declaration of Helsinki of 1964 as revised in 2013. The study participants provided full and informed consent before inclusion. Due to the retrospective nature of this study, it was granted an exemption in writing by the local ethics committee. The Strobe Checklist was followed writing this paper.

### Statistical Analysis

The main outcome of this study was to evaluate the success rate of implant-supported SC and FPD using ASC; the secondary outcome was to evidence the effect of several variables on this rate.

The statistical analysis was based on the success rates of the reconstructions. At each follow-up, each restoration was defined as successful if it was still in use and if no complications had occurred. When one of these two events occurred (either failure of the restoration or a mechanical/biological complication) the patient was dropped from the study, and the restoration was considered as unsuccessful. If a patient missed a follow-up appointment, the restoration was considered as censored, and the patient was removed from the study.

The survival time of a restoration was defined as the period between baseline (month of delivery) and either the last follow-up examination or, in the occurrence of a complication, the month of diagnosis. The time-dependent success rates of the restorations were calculated by Kaplan–Meier survival analysis.

Categorical data were analyzed as absolute and relative frequency. A Cox model was created inserting the variables of immediate interest (design of the restoration, type of restorations, position of the restoration, and condition of the antagonist tooth) to evaluate their effect on the restoration’s success rates.

A power analysis was conducted and, given the design of our study and expecting an 8% probability of the researched event, in order to reach a statistical power of 80% for a Cox proportional hazards model, expecting a hazard ratio of 5, given a 0.6 standard deviation, and at a significance level of 0.05, a minimum sample size of 96 subjects was required.

The statistical analysis and the images were obtained using statistical software (STATA 17, StataCorp LLC, College Station, TX, USA).

## 3. Results

From January 2017 to December 2019, 105 subjects were included in the present study and treated with 105 implant-supported prostheses (either an SC or an FPD); the mean follow-up time was 33 months with a standard deviation of 9.93, with a minimum observation time of 5 months and a maximum of 53 months. A total of 52 restorations were MZR, and the remaining 53 restorations were PVR.

The mean age of the sample was 52.93 (Standard deviation = 9.53); other demographical characteristics of the sample are reported in Table 1.

Three implants failed in the first year after prosthetic-loading, while two PVR prostheses had a chipping of the veneering ceramic, and two SC (one PVR and one MZR) had to be tightened as the restoration came loose.

### Success Rate

Almost half of the sample (52 subjects, 49%) was followed for 36 months or more, while only seven patients (6.7%) were followed for more than 4 years. No subjects reached the 5 years mark (Table 2).

**Figure 4:**
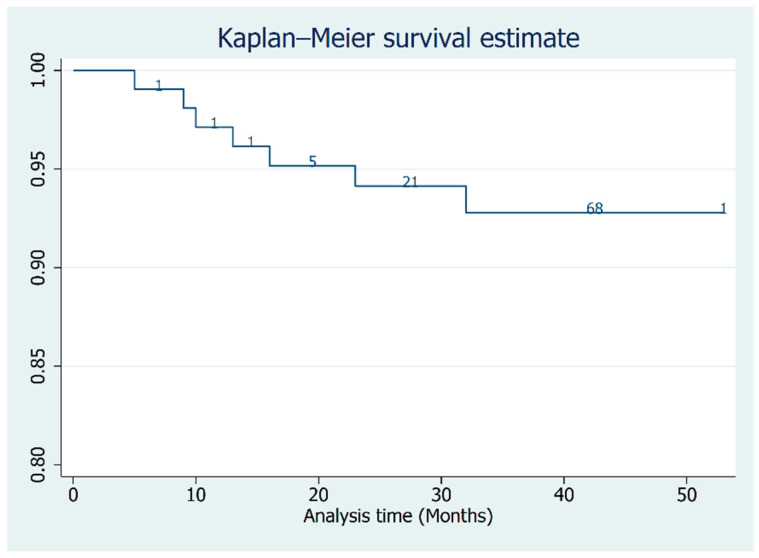
The Kaplan–Meier curve of the entire sample; the number of patients censored at each follow-up are reported in the graph.

After one year a survivor function of 0.98% with a confidence interval (CI) of 0.93–0.99% was observed. After 2 years it dropped to 0.94% (CI 0.87–0.97%). At the 46-months follow-up a success rate of 0.92% (CI 0.85–0.96%) was reached. The Kaplan–Meier function (Figure 4) shows how the success rate slowly decreased throughout the follow-up period, taking in consideration the patients that were censored from the study.

Seven restorations did not provide a successful outcome; six unsuccessful restorations were located in the posterior region.

**Figure 5:**
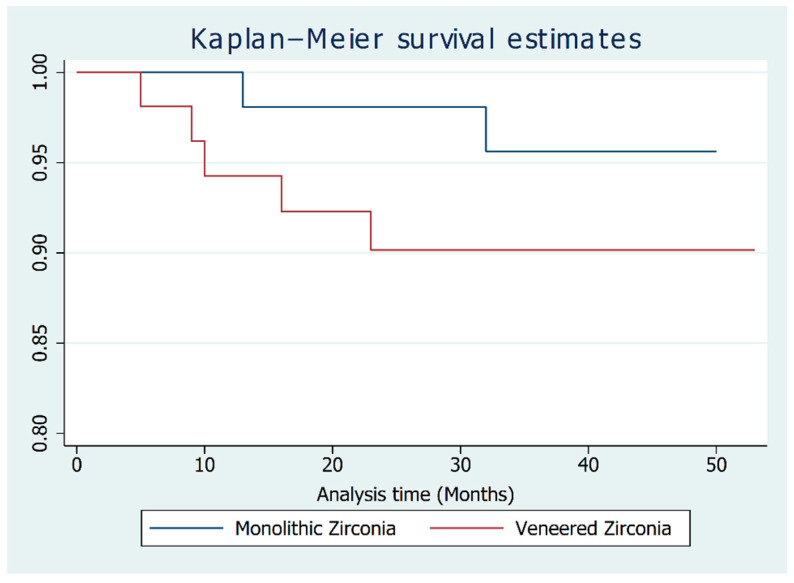
The Kaplan–Meier curves for both restorative materials.

To highlight the effects the application of a partial veneer has on the success rate of these restorations, a Kaplan–Meier function, stratified according to the material, is presented in Figure 5. The two curves diverge after only 4 months, indicating a higher success rate for the monolithic restorations throughout the whole follow-up period. This was expected, as monolithic restorations tend to have a lower frequency of mechanical complications.

After 47 months, the success rates of MZR and PVR prostheses were, respectively, 0.95 and 0.90 (Table 3). Following the application of a Cox Model, monolithic restoration had a 0.16-fold reduced risk of complications (Hazard Ratio = 6.04) when compared to partially veneered one. This effect proved to be significant (*p* = 0.039); therefore, the null hypothesis had to be rejected. The results of the model are presented in Table 4.

MZR with ASCs have a higher chance of success than PVR. Anterior restorations had a 0.15-fold reduced risk of complications (Hazard Ratio = 6.59), but this effect was not statistically significant (*p* = 0.09). None of the other variables had a statistically significant effect.

## 4. Discussion

The present study reported on the success rate of screw-retained FPD employing ASC. At the 2 years follow-up, a success rate of 94% was observed; this is in line with what other studies reported on FPD not employing ASC [26,27,28], hinting that this retentive system does not reduce the efficacy of these kind of restorations.

ASC provides several advantages as it allows the application of screw-retained restorations in clinical situation where, given the inclination of the implant fixture, one would have been forced to use a cement-retained restoration. As Edmondson et al. [16] have said, thanks to ASC, almost every implant-supported SC in the anterior region can be screw-retained. This is of paramount importance given the innate advantages of this fixation system when compared to cement-retained restorations, the difficulties in retrieving the restoration, and the risks of cement-related peri-implantitis.

The main concern regarding this fixation system is the reduced preload that can be applied to the retentive screw; this might lead to a higher rate of loosening of the restoration, especially in the posterior region [23]. Their application in zirconia abutments also showed a higher rate of abutment fracture [20]. 

Only a few in vivo reports have reported on these restorations, and their results hint that this system can provide satisfying success and survival rates, even if at short follow-ups [21,29].

This study is the first to apply ASC to a significant number of restorations and to evaluate their efficacy at a longer follow-up. Half of our restorations were located in the posterior region, where occlusal forces are much higher than in the anterior region; this shows that even considering the intrinsic geometrical characteristics of fixating screws and screwdriver, the risk of the restoration coming loose is still reduced as we only observed two fixating screws loosening. Our first Kaplan–Meier model shows that these restorations have a success rate similar to all-ceramic, conventional screw-retained, implant-supported restorations; as for these restorations, authors have reported a success rate of approximately 95% [30].

Monolithic zirconia is nowadays commonly used for rehabilitations located in the posterior zone of the arch as this material can offer high mechanical strength, as reported in many in vitro studies [31]. A few in vivo reports have also observed that these restorations are less prone to mechanical complications and failure than conventional veneered restorations and partially veneered restorations [32,33].

Our paper reached the same conclusions: following our Kaplan–Meier model, monolithic restorations had a higher success rate throughout the whole follow-up period, and the Cox model confirmed this finding.

The null hypothesis was therefore rejected: monolithic restorations with ASC have higher success rates than partially veneered ones, even when accounting for other factors such as the location of the restorations in the dental arch and the condition of the antagonist tooth.

This observation is especially important given that most monolithic restorations were in the posterior zone, where restorations are subjected to higher forces and therefore more prone to complications. This effect was also observed in our analysis, even if not statistically significant.

Given the design of our retrospective study, some flaws must be reported. First and foremost, these observations are limited to one specific type of abutment and may not be applicable to other brands. Additionally, while the mean follow-up was of 33 months, the distribution was particularly diversified including different types of restorations, and from our populations only 56 patients were followed for 3 years or more and most of the complications occurred before this time mark; there is therefore a possibility that more complications were not recorded as these patients did not attend recall appointments. Further research is expected to directly relate the survival rate to the entity of angulation of the screw access channel, and to observe the feasibility of ASC in zirconia abutments.

Implant-supported FPD employing ASC offers a success rate similar to conventional, screw-retained FPD. Monolithic restorations, thanks to their higher mechanical strength, have a lower failure rate. More prospective, long-term studies are needed to confirm the findings of our study.

## Figures and Tables

**Table 1:** Characteristics of the sample.

Variable	Absolute Frequency (Relative Frequency)
**Sex**	
**Male**	52 (49.52%)
**Female**	53 (50.48%)
**Design of Restoration**	
**Monolithic Zirconia**	52 (49.52%)
**Partially veneered Zirconia**	53 (50.48%)
**Type of restoration**	
**FPD**	51 (48.57%)
**SC**	54 (51.43%)
**Condition of Antagonist Tooth**	
**Natural Tooth**	58 (55.24%)
**Tooth supported FPD**	26 (24.76%)
**Implant supported FPD**	12 (11.43%)
**Missing antagonist**	4 (3.81%)
**RPD**	5 (4.76%)

FPD = Fixed Partial Denture, SC = Single Crown, RPD = Removable partial denture.

**Table 2:** Survivor functions at major time points of the entire sample.

Time (Months)	At Risk	Fail	Lost	Survivor Function	95% Confidence Interval	
5	105	1	0	0.99	0.93	0.99
7	104	0	1	0.99	0.93	0.99
9	103	2	1	0.98	0.92	0.99
12	100	2	3	0.97	0.91	0.99
18	100	1	6	0.95	0.88	0.97
24	88	0	16	0.94	0.87	0.97
30	72	1	19	0.94	0.87	0.97
36	52	0	35	0.92	0.85	0.96
42	17	0	10	0.92	0.85	0.96

**Table 3:** Survivor functions at major time points according to the different materials adopted.

Time	Survivor Function	
	Monolithic Zirconia	Partially Veneered Zirconia
12 Months	1.0	0.94
18 Months	0.98	0.92
36 Months	0.95	0.90
47 Months	0.95	0.90
53 Months	–	0.90

**Table 4:** The results of the Cox model.

Variables	Hazard Ratio	*p*-Value	95% Confidence Interval
Design of restoration	–	–	–
Stratified Zirconia	6.044	0.039	1.102–37.626
Type of restoration	–	–	–
Single Crown	0.420	0.279	0.087–2.021
Position of restoration	–	–	–
Posterior Restoration	6.592	0.099	0.700–62.043
Condition of antagonist tooth	–	–	–
Tooth Supported FPD	1.148	0.876	0.201–6.535
Implant Supported FPD	0.845	0.895	0.070–10.127
RPD	<0.001	1	–
Missing antagonist	<0.001	1	–

## Data Availability

The data presented in this study are available on request from the corresponding author. The data are not publicly available due to privacy restriction.
